# Examining differences between mass, multiple, and single-victim homicides to inform prevention: findings from the National Violent Death Reporting System

**DOI:** 10.1186/s40621-021-00345-7

**Published:** 2021-08-09

**Authors:** Katherine A. Fowler, Rachel A. Leavitt, Carter J. Betz, Keming Yuan, Linda L. Dahlberg

**Affiliations:** 1 National Center for Injury Prevention and Control, Division of Violence Prevention, Centers for Disease Control and Prevention, Atlanta, GA USA; 2 National Center for Injury Prevention and Control, Division of Injury Prevention, Centers for Disease Control and Prevention, Atlanta, GA USA

**Keywords:** Violence prevention, Homicide, Firearm violence, Mass shootings, Mass homicide, Violence perpetration, Intimate partner violence, Suicide, Family violence, Surveillance, Injury

## Abstract

**Background:**

Multi-victim homicides are a persistent public health problem confronting the United States. Previous research shows that homicide rates in the U.S. are approximately seven times higher than those of other high-income countries, driven by firearm homicide rates that are 25 times higher; 31% of public mass shootings in the world also occur in the U.S.. The purpose of this analysis is to examine the characteristics of mass, multiple, and single homicides to help identify prevention points that may lead to a reduction in different types of homicides.

**Methods:**

We used all available years (2003–2017) and U.S. states/jurisdictions (35 states, the District of Columbia, and Puerto Rico) included in CDC’s National Violent Death Reporting System (NVDRS), a public health surveillance system which combines death certificate, coroner/medical examiner, and law enforcement reports into victim- and incident-level data on violent deaths. NVDRS includes up to 600 standard variables per incident; further information on types of mental illness among suspected perpetrators and incident resolution was qualitatively coded from case narratives. Data regarding number of persons nonfatally shot within incidents were cross-validated when possible with several other resources, including government reports and the Gun Violence Archive. Mass homicides (4+ victims), multiple homicides (2-3 victims) and single homicides were analyzed to assess group differences using Chi-square tests with Bonferroni-corrected post-hoc comparisons.

**Results:**

Mass homicides more often had female, child, and non-Hispanic white victims than other homicide types. Compared with victims of other homicide types, victims of mass homicides were more often killed by strangers or someone else they did not know well, or by family members. More than a third were related to intimate partner violence. Approximately one-third of mass homicide perpetrators had suicidal thoughts/behaviors noted in the time leading up to the incident. Multi-victim homicides were more often perpetrated with semi-automatic firearms than single homicides. When accounting for nonfatally shot victims, over 4 times as many incidents could have resulted in mass homicide.

**Conclusions:**

These findings underscore the important interconnections among multiple forms of violence. Primary prevention strategies addressing shared risk and protective factors are key to reducing these incidents.

## Background

Multi-victim homicides are a persistent public health problem confronting the United States. Homicide is a leading cause of death in the U.S., particularly for people under age 45 years, (Centers for Disease Control and Prevention, 2005) with 19,141 deaths for the nation in 2019 (age-adjusted rate: 6.0 per 100,000). Homicide rates in the U.S. are approximately seven times higher than those of other high-income countries, driven by firearm homicide rates that are 25 times higher (Grinshteyn & Hemenway, 2019). Compared with other high-income countries, the US has the most public mass shootings by far, accounting for 31% globally, despite only accounting for 5% of the global population (Lankford, 2016).

Firearm homicides and assaults – whether single victim or multi-victim – are traumatic events that can affect the sense of safety and security of entire communities. Studies examining the aftermath of multi-victim homicides report increased rates of negative mental health outcomes, including Post-Traumatic Stress Disorder (PTSD), major depression, alcohol dependence, and anxiety disorders among survivors(North et al., 1997; North et al., 2002; Lowe & Galea, 2017), and decreased feelings of safety and increased fear among communities (Lowe & Galea, 2017; Fullerton et al., 2019), even including those living far beyond affected communities (Lowe & Galea, 2017).

Public mass shootings with large numbers of victims are the most frequently represented form of multi-victim homicide in national media portrayals,(Duwe, 2000) but mass homicides are defined differently across research, government, and media organizations. The Federal Bureau of Investigation (FBI) defines mass homicides as having four or more homicide victims within the same incident (not including the perpetrator), typically in a single location, not defined by the type of weapon used.(Federal Bureau of Investigation, 2008) Reports from different organizations have tended to more narrowly focus on mass shootings, as a particular public concern and challenge for law enforcement responders. (Follman et al., 2020; Everytown for Gun Safety, n.d.-a; Gun Violence Archive, n.d.; Blair et al., 2014; O’Neill et al., 2016) These reports use the FBI criterion of four or more victims but have differed in whether the number of fatalities only (Follman et al., 2020; Everytown for Gun Safety, n.d.-a), or both fatally and nonfatally shot (O’Neill et al., 2016) define an incident. Another difference is whether all mass shootings (Everytown for Gun Safety, n.d.-a; Gun Violence Archive, n.d.) or only those that occur in public locations (Follman et al., 2020) are counted. Others report on “active shooter” incidents, focusing on the law enforcement response to incidents involving a shooter trying to kill people in a confined and populated area. (Blair et al., 2014; O’Neill et al., 2016) In a comparison of four major databases of mass homicide (Everytown for Gun Safety, Gun Violence Archive, FBI’s Supplemental Homicide Reports, and Mother Jones), Booty et al. (2019) found that in one data year, the number of incidents included in each database ranged from 11 to 346 incidents, with only two incidents included in all four databases (Booty et al., 2019). These differences in definition for mass homicide incidents create difficulty in understanding the burden and prevention of these incidents.

In addition to varying and often restrictive definitions, research on mass homicides faces several other limitations. These include focusing only on certain victim or offender types (Fox & Levin, 2015); excluding non-random incidents, such as those related to crime or relationship problems (Taylor, 2018); reliance on convenience samples such as media accounts; inclusion of only firearm homicides or only those that occur in public; and lack of systematic comparison with other types of homicides that may identify unique characteristics of these incidents. These limitations may leave important points of prevention unstudied.

The purpose of this study is to use public health surveillance data that integrates information from several investigative data sources to analyze the characteristics of mass homicides, and to compare them with multiple and single homicides to help identify prevention points that may lead to a reduction in different types of homicides. This study applies a broad definition that is not restricted by weapon type, location, or motivation, with the goal of describing the full public health burden.

## Methods

### Case definitions

In keeping with standard definitions within NVDRS, homicides are defined as fatal injuries resulting from the intentional use of force. Homicide incidents are defined as one or more related deaths meeting the case definition for homicide that occurred within the same 24-hour period. The determination regarding whether deaths are related and therefore part of the same incident in NVDRS is made based on information linking the deaths found in the investigative reports used in data abstraction (see below). (CDC, 2018) Consistent with FBI definitions, mass homicides are defined as having four or more victims, not including perpetrators. Multiple homicides are defined as having two to three victims other than the perpetrator. Single homicides have one victim. Cases are defined by number fatally injured, regardless of motivation, and encompass all victim-suspect relationships types (e.g., family, stranger, intimate partner) and weapon types (not limited to firearms).

### Data source

We used data from all available years at the time of the analysis (2003–2017) in CDC’s National Violent Death Reporting System (NVDRS), which combines death certificate, coroner/medical examiner (C/ME), and law enforcement (LE) reports into victim- and incident-level data on violent deaths. (Petrosky et al., 2020) In brief, trained data abstractors compile information from these records into standardized variables in the NVDRS web-based system using CDC guidance and definitions. Abstractors compose two case narratives for each incident as well, one from the perspective of the law enforcement report, and one from the perspective of the coroner/medical examiner report. The case narratives summarize and describe the incident and help to provide context.

All 50 U.S. states, the District of Columbia and Puerto Rico are currently funded to participate in NVDRS, but at the time of this study several of the most recently funded states had not yet completed a data collection cycle and therefore are not included. States and jurisdictions were first funded to participate in NVDRS in different years. Data for this study comes from the following 37 states/jurisdictions: Maryland, Massachusetts, New Jersey, Oregon, South Carolina, and Virginia (2003–2017); Alaska, Colorado, Georgia, North Carolina, Oklahoma, Rhode Island, and Wisconsin (2004–2017); Kentucky, New Mexico, and Utah (2005–2017); Ohio (2010–2017); Michigan (2014–2017); Arizona, Connecticut, Illinois, Indiana, Iowa, Kansas, Maine, Minnesota, New Hampshire, New York, Pennsylvania, Vermont, and Washington (2015–2017); Hawaii (2015–2016); California, Delaware, West Virginia, the District of Columbia, and Puerto Rico (2017). Illinois, Pennsylvania, and Washington collected data on ≥80% of violent deaths in their state, in accordance with requirements under which these states were funded. Data for California are for violent deaths that occurred in four counties (Los Angeles, Sacramento, Shasta, and Siskiyou). All other states and jurisdictions provided data from all areas.

NVDRS has a number of unique advantages as a data source for information about multi-victim homicides in comparison with other federal databases. For example, although CDC’s National Vital Statistics System (NVSS) captures nationwide information about deaths due to homicide, its information is derived completely from death certificates, and there is no way to link individual deaths that occurred in the same incident. The FBI‘s Supplementary Homicide Reports (SHR) gather additional details for homicides such as the age, sex, race, and ethnicity of both the victim and the offender, the weapon used in the homicide, the circumstances surrounding the offense, and the relationship of the victim to the offender as part of its Uniform Crime Reporting Program, while the National Incident-Based Reporting System (NIBRS) collects information about the number of victims in an incident and basic information about weapon types and victim-offender relationships; however, the reporting for both is voluntary and not all states and jurisdictions participate.

In contrast, NVDRS now collects nationwide data (although fully national data were not available at the time of the study) and participating states can easily identify multi-victim incidents since multiple types of investigative reports provide data, and the level of detail collected in the system about these incidents greatly surpasses that of NVSS, SHR, or NIBRS. NVDRS collects information on up to 600 variables per victim, including detailed information on injury characteristics; demographics of victims and suspects; circumstances that precipitated the incident (including those specific to homicides); victim toxicology records; mental health of victims and suspects; weapons used to inflict the fatal injury; characteristics of firearms used to inflict the fatal injuries; and the number of victims, suspects, and manners of death (e.g., homicide, suicide, legal intervention) within incidents. Further, unlike media-based data sources, NVDRS uses de-identified information from official investigative reports and is ideal for comparing mass homicides versus a representative pool of all other homicide incidents due to the scope of the system.

This analysis was conducted using two NVDRS datasets: One was the standard NVDRS Restricted Access Database file, which captures case-level data on all variables in NVDRS. The other was an incident-level dataset developed by the authors specifically to consolidate information about incidents with more than one homicide. The incident-level dataset consolidates all information about incident characteristics, circumstances, and suspects for the incident into one case so that information such as precipitating circumstances and suspect demographics will not be overrepresented in the data as they would if repeated for each victim. This avoids over-weighting incidents with high victim counts when comparing incidents with one another.

Per CDC requirements, VDRS programs must have circumstance information from either coroner/medical examiner reports or law enforcement reports for at least 50% of cases to be included in the national dataset. However, VDRS programs often far exceed this requirement; according to recent estimates, 87.5% of suicides, homicides, and legal intervention deaths in NVDRS had circumstance data from C/ME and/or LE reports. In addition, core variables that represent demographic characteristics (e.g., age, sex, and race/ethnicity) and manners of death were complete for almost 99% of NVDRS cases (Petrosky et al., 2020).

From all data captured by NVDRS, variables that were considered the most relevant to comparisons of homicide types were selected for analysis. Victim demographics and victim-suspect relationship were analyzed at the victim level to reflect the public health burden of different types of homicides on individuals. To aggregate characteristics of incidents as a whole, incident-level data were analyzed for suspect demographics; mental health and suicidal behavior of suspected perpetrators; location type; primary weapons used; number of persons nonfatally shot in the incident; precipitating circumstances; and incident resolution.

### Qualitative methods

#### Coding additional variables

Additional variables were coded from case narratives according to definitions established for this study. These variables included: type of mental illness among suspects (for incidents where the perpetrator’s mental illness reportedly precipitated the incident), and case resolution (e.g., how the incident ended, mass homicides only). All mass homicide cases were double-coded for ‘case resolution’ by five of the authors, with discrepancies resolved by group consensus.

In NVDRS, a standard variable is available for abstractors to select if the suspect’s attack on the victim is believed to be the direct result of a mental illness (of any type). (Petrosky et al., 2020) To further understand the types of mental illness among suspects, the authors developed standardized coding guidance that categorized types of mental illness into the following groups and outlined the criteria for each: attention deficit hyperactivity disorder (ADHD), anxiety, bipolar disorder, dementia, depression, personality disorder(s), post-traumatic stress disorder (PTSD), schizophrenia spectrum, unspecified psychosis, and unspecified mental illness. These groupings are based on disorders and syndromes listed in the Diagnostic and Statistical Manual of Mental Disorders, Fifth Edition (DSM-5), and parallel categories typically captured for victim mental health problems in NVDRS."Unspecified psychosis,” was added to these categories to capture cases where clear symptoms of psychosis (e.g., hallucinations, delusions) were described but schizophrenia (or a related disorder, such as schizoaffective disorder) was not specifically identified.

Type of suspect mental illness was double-coded for all 12 mass and all 95 multiple homicide incidents indicated to be directly precipitated by the suspect’s mental illness by the standard NVDRS variable. Two hundred single homicides indicated to be directly precipitated by the suspect’s mental illness were also double-coded; at this point inter-rater reliability was > 95%, so raters then single-coded the remaining 656 single homicide cases indicated as directly precipitated by the suspect’s mental illness.

#### Identifying and validating number nonfatally shot

NVDRS has a standard variable for number of nonfatally shot persons within an incident. However, due to variability in completeness of sources, some cases with a number of nonfatally shot persons may erroneously have missing data for this variable in NVDRS. Therefore, we cross-validated cases with several other databases that track this information: the Gun Violence Archive (GVA), (Gun Violence Archive, n.d.) Mother Jones’ Mass Shootings Database (Follman et al., 2020), the Everytown for Gun Safety mass shootings report (Everytown for Gun Safety, n.d.-a), the NYPD active shooter report (O’Neill et. al., 2016) and its updates/appendices, and the FBI active shooter report (Blair et al., 2014). Multiple databases were used due to differences in coverage/case definitions, and limitations of the different sources.

Cases were matched on dates and locations of occurrence and then reviewed if NVDRS differed from one or more matches. Across all years, 3% of NVDRS incidents (*n* = 2671) had a match in one (99%) or more (1%) databases (34% of mass homicides; 7% of multiple homicides; 3% of single homicides). The vast majority of these were matched with GVA (> 99%), due to its broad coverage; for the years covered by both NVDRS and GVA, 6% of single homicides, 12% of multiple homicides, and 41% of mass homicides had a match. Among those cases that had a match, 60% had the same number of nonfatally shot persons in NVDRS (77% of mass homicides, 59% of multiple homicides; 59% of single homicides). Values that did not match NVDRS were reviewed by the co-authors, and the non-NVDRS database was found to be more accurate in almost all cases. The most common scenario leading to source disagreement was that NVDRS had zero persons nonfatally shot on record, while the validating source indicated one or more persons nonfatally shot (85% of disagreements). Most cases with nonfatally shot persons included homicides as part of the NVDRS incident. However, 643 single suicide incidents, and 275 legal intervention deaths had nonfatally shot persons as part of the incident as well.

### Statistical methods

Victim, suspect, and incident characteristics were compared by type of homicide (mass, multiple, and single homicides). Given the multi-level data structure, bivariate statistical techniques were best suited to this analysis. We used traditional Pearson chi-square tests to assess the incident level variables. Rao–Scott chi-square tests were applied to victim and suspect variables to account for the clustering of victims/suspects within incidents. Significant chi-squared results (*p* < 0.05) were further examined with post-hoc pairwise comparisons, and Bonferroni corrections were applied to account for multiple comparisons.

## Results

**Fig. 1:**
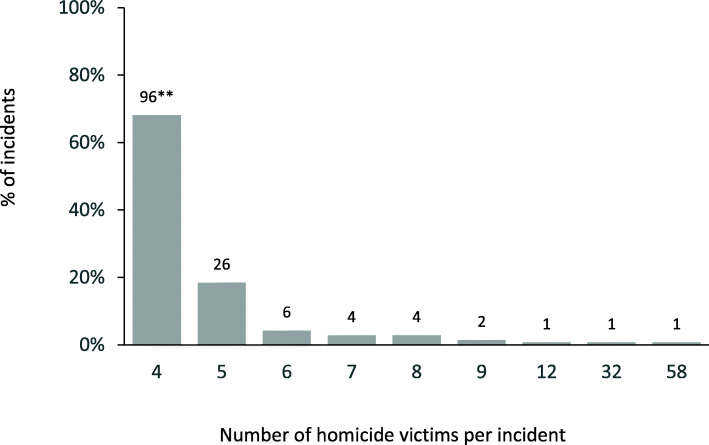
Distribution of the number of victims in mass homicide incidents (*N* = 141), National Violent Death Reporting System, 2003–2017*. * All 50 U.S. states, the District of Columbia and Puerto Rico are currently funded to participate in NVDRS, but at the time of this study several of the newer states/jurisdictions had not yet completed a data collection cycle and therefore are not included. States and jurisdictions were first funded to participate in NVDRS in different years. Data for this study comes from the following 37 states/jurisdictions: Maryland, Massachusetts, New Jersey, Oregon, South Carolina, and Virginia (2003–2017); Alaska, Colorado, Georgia, North Carolina, Oklahoma, Rhode Island, and Wisconsin (2004–2017); Kentucky, New Mexico, and Utah (2005–2017); Ohio (2010–2017); Michigan (2014–2017); Arizona, Connecticut, Illinois, Indiana, Iowa, Kansas, Maine, Minnesota, New Hampshire, New York, Pennsylvania, Vermont, and Washington (2015–2017); Hawaii (2015–2016); California, Delaware, West Virginia, the District of Columbia, and Puerto Rico (2017). Illinois, Pennsylvania, and Washington collected data on ≥80% of violent deaths in their state, in accordance with requirements under which these states were funded. Data for California are for violent deaths that occurred in four counties (Los Angeles, Sacramento, Shasta, and Siskiyou). **Number above bar represents the number of incidents with the number of victims indicated in corresponding column on x-axis (e.g., *N* = 96 out of the total of 141 mass homicide incidents had 4 victims)

There were 728 victims of mass homicide killed in 141 incidents (range 4-58 victims, Fig. 1; median, mode *n* = 4); 7112 victims of multiple homicide killed in 3439 incidents; and 74,623 victims of single homicide in NVDRS between 2003 and 2017 (Tables 1 and 2).

**Table 1:** Victim and suspect characteristics by homicide type, National Violent Death Reporting System, 2003–2017*

	Victims				Suspects/Perpetrators**			
	Mass homicide n(%)	Multiple homicide n(%)	Single homicide n(%)	Chi-square ***p***-value	Mass homicide n(%)	Multiple homicide n(%)	Single homicide n(%)	Chi-square p-value
Total***	728	7,112	74,623		154	3,651	68,530	
**Sex**								
Male	351 (48.2) ^a,b^	4,611 (64.8) ^c^	59,351 (79.6)	< 0.001	133 (95.0)	3,067 (92.9) ^c^	54,015 (89.7)	< 0.001
Female	377 (51. 8) ^a,b^	2,501 (35.2) ^c^	15,251 (20.4)	< 0.001	7 (5)	236 (7.2) ^c^	6,188 (10.3)	< 0.001
**Age group (years)**								
0–10	116 (16.0) ^a,b^	550 (7.8) ^c^	3,470 (4.7)	< 0.001	0(.)	0(.)	30 (0.1)	N/A
11-17	74 (10.2) ^a,b^	451 (6.4) ^c^	3,081 (4.1)	< 0.001	5 (4.0)	137 (5.0) ^c^	3,115 (6.9)	0.001
18-24	132 (18.2)	1,596 (22.5)	17,538 (23.5)	0.05	31 (24.6)	868 (32.0)	15,409 (34.2)	0.02
25-34	131 (18.0) ^a,b^	1,755 (24.7) ^c^	20,096 (27.0)	< 0.001	42 (33.3)	857 (31.6) ^c^	12,772 (28.4)	0.003
35–44	87 (12.0) ^b^	993 (14.0) ^c^	12,540 (16.8)	< 0.001	26 (20.6)	450 (16.6) ^c^	6,476 (14.4)	0.002
45-54	83 (11.4)	788 (11.1)	8,978 (12.1)	0.11	17 (13.5)	261 (9.6)	4,106 (9. 1)	0.20
55–64	54 (7.4)	506 (7. 1)	5,002 (6.7)	0.43	5 (4.0)	93 (3.4)	1,919 (4.3)	0.11
65+	50 (6.9)	459 (6.5) ^c^	3,803 (5. 1)	0.001	0(.)	47 (1.7) ^c^	1,198 (2.7)	N/A
**Race/Ethnicity**								
Non-Hispanic white	407 (55.9) ^a,b^	2,648 (37.2) ^c^	21,943 (29.4)	< 0.001	67 (43.5) ^a,b^	1,020 (27.9) ^c^	14,869 (21.7)	< 0.001
Non-Hispanic black	181 (24.9) ^a,b^	3,146 (44.2) ^c^	39,806 (53.3)	< 0.001	43 (27.9) ^a,b^	1,456 (39.9) ^c^	29,177 (42.6)	< 0.001
Non-Hispanic American Indian/Alaska Native	13 (1.8)	136 (1.9)	1,571 (2.1)	0.75	2 (1.3)	38 (1.0)	864 (1.3)	0.55
Non-Hispanic Asian/Pacific Islander	23 (3.2)	167 (2.4) ^c^	1,158 (1.6)	< 0.001	6 (3.9) ^a,b^	52 (1.4) ^c^	524 (0.8)	< 0.001
Hispanic***	98 (13.5)	964 (136)	9,623 (12.9)	0.60	14 (9.1)	315 (8.6)	5,063 (7.4)	0.05
**Victim-Suspect Relationship**								
Stranger	138 (19.0) ^a,b^	515 (7.2)	4,673 (6.3)	< 0.001				
Acquaintance/friend	98 (13.5)	776 (10.9) ^c^	9,491 (12.7)	0.25				
Child	74 (10.2) ^b^	483 (6.8) ^c^	2,028 (2.7)	< 0.001				
Other relative/family member	66 (9.1) ^a,b^	339 (4.8) ^c^	2,009 (2.7)	< 0.001				
Spouse/intimate partner	35 (4.8) ^a,b^	530 (7.5) ^c^	7,899 (10.6)	< 0.001				
Other person known to victim	30 (4.1) ^a^	635 (8.9) ^c^	5,209 (7.0)	< 0.001				
Parent	20 (2.8)	310 (4.4) ^c^	1,477 (2.0)	< 0.001				
Other intimate partner involvement	12 (1.7)	88 (1.2)	778 (1.0)	0.32				
Rival gang member	8 (1.1)	93 (1.3) ^c^	679 (1.0)	0.15				
Victim was law enforcement officer injured in the line of duty	3 (0.4)	30 (0.4) ^c^	138 (0.2)	0.002				
Current/former work relationship	0(.)	50 (0.7) ^c^	288 (0.4)	N/A				
Other/Unknown relationship	244 (33.5)	3,263 (45.9) ^c^	39,954 (53.5)	< 0.001				
Suspect/Perpetrator Characteristics****								
**Mental health problem directly related to incident** ^d^					13 (10.3) ^a,b^	132 (4.7) ^c^	1,226 (2.2)	< 0.001
**Type of mental health problem** ^e^								
Unspecified psychosis					4 (30 8) ^b^	16 (12.2)	102 (8.3)	0.01
Unspecified mental illness					3 (23.1)	39 (29.8)	358 (29.2)	0.88
Bipolar disorder					2 (15.4)	11 (8.4)	95 (7.8)	0.58
ADHD					2 (15.4) ^b^	3 (2.3)	15 (1.2)	0.01
Anxiety					2 (15.4)	2 (1.5)	27 (2.2)	0.04
Schizophrenia spectrum					1 (7.7)	15 (115)	143 (11.7)	0.90
Depression					0 (0)	18 (13.7) ^c^	66 (5.4)	< 0.001
PTSD					0 (0)	5 (3.8)	33 (2.7)	0.63
Dementia					0 (0)	1 (0.8)	48 (3.9)	0. 14
Personality disorder					0 (0)	1 (0.8)	4 (0.3)	0.71
**Suicidal thoughts or behaviors** ^d^					39 (30. 1) ^a,b^	490 (17.3) ^c^	3,540 (6.4)	< 0.001

**Abbreviations:** *PTSD* posttraumatic stress disorder *ADHD* attention deficit hyperactivity disorder *N/A* not applicable

*All 50 U.S. states, the District of Columbia and Puerto Rico are currently funded to participate in NVDRS, but at the time of this study several of the newer states/jurisdictions had not yet completed a data collection cycle and therefore are not included. States and jurisdictions were first funded to participate in NVDRS in different years. Data for this study comes from the following 37 states/jurisdictions: Maryland, Massachusetts, New Jersey, Oregon, South Carolina, and Virginia (2003–2017); Alaska, Colorado, Georgia, North Carolina, Oklahoma, Rhode Island, and Wisconsin (2004–2017); Kentucky, New Mexico, and Utah (2005–2017); Ohio (2010–2017); Michigan (2014–2017); Arizona, Connecticut, Illinois, Indiana, Iowa, Kansas, Maine, Minnesota, New Hampshire, New York, Pennsylvania, Vermont, and Washington (2015–2017); Hawaii (2015–2016); California, Delaware, West Virginia, the District of Columbia, and Puerto Rico (2017). Illinois, Pennsylvania, and Washington collected data on ≥80% of violent deaths in their state, in accordance with requirements under which these states were funded. Data for California are for violent deaths that occurred in four counties (Los Angeles, Sacramento, Shasta, and Siskiyou)

**Suspect information is reported at the incident level. Percentages are based on the number of incidents with known suspect information: mass (126), multiple (2,826), single (55,468). The following sentence can be used as a guide for interpreting victim-suspect relationship: “The victim is the ____________ of the suspect”. For example, when a parent kills a child, the relationship is “Child” not “Parent” (“The victim is the child of the suspect”). Please note that this sentence is intended to be a general guide. However, some relationships may not be captured by this sentence (e.g., other person known to victim; victim was law enforcement officer killed in the line of duty). The victim-suspect relationship was known for 66% of mass homicide victims, 54% of multiple homicide victims, and 46% of single homicide victims

***Includes persons of any race﻿

****Numbers may not sum to total because of missing values

^a^ Statistically significant difference (p < .05) of the prevalence of the characteristic between mass versus multiple homicide

^b^ Statistically significant difference (*p* < .05) of the prevalence of the characteristic between mass versus single-victim homicide

^c^ Statistically significant difference (p < .05) of the prevalence of the characteristic between multiple versus single-victim homicide

^d^ Applied to any suspect in the incident

^e^ Denominator includes only incidents indicated as directly attributable to mental illness on the part of the suspect. Mental illness categories are not mutually exclusive

**Table 2:** Incident characteristics* by homicide type, National Violent Death Reporting System, 2003–2017**

	Incidents			
	Mass homicide n(%)	Multiple homicide n(%)	Single homicide n(%)	Chi-square p-value
**Total**	141	3,439	74,623	
**Location of Incident**				
Private home/apartment	95 (67.4) ^b^	2,019 (58.7) ^c^	35,113 (47.1)	< 0.001
Public location	29 (20.6) ^a,b^	1,033 (30.0) ^c^	35,171 (47.1)	< 0.001
More than one location	16 (11.4) ^b^	328 (9.5) ^c^		< 0.001
Unknown	1 (0.7) ^b^	59 (1.7) ^c^	4,339 (5.8)	< 0.001
**Primary weapon used to inflict fatal injuries**				
Firearm	104 (74.3) ^a^	2,810 (82.3) ^c^	49,509 (69.2)	< 0.001
Other	36 (25.7) ^a^	604 (17.7) ^c^	22,062 (30.8)	< 0.001
**Number of weapons used to inflict fatal injuries**				
1	120 (85.7) ^a,b^	3,208 (93.5) ^c^	72,460 (99.6)	< 0.001
2	17 (12.1) ^a,b^	210 (6 1) ^c^	313 (0.4)	< 0.001
3+	3 (2.1) ^b^	14 (0.4) ^c^	0 (0)	< 0.001
**One or more nonfatally shot**	30 (21.3) ^b^	561 (16.3) ^c^	6,008 (8.1)	< 0.001
**Number nonfatally shot**				
0	107 (75.9) ^b^	2,774 (80.7) ^c^	62,525 (83.8)	< 0.001
1	14 (9.9)	398 (11.6) ^c^	4,695 (6.3)	< 0.001
2	5 (3.6)	95 (2.8) ^c^	920 (1.2)	< 0.001
3+	11 (7. 8) ^a,b^	68 (2.0) ^c^	393 (0.5)	< 0.001
Unknown	4 (2. 8)	104 (3.0) ^c^	6,090 (8.2)	< 0.001
Perpetrated by more than one suspect***	23 (18.3)	563 (20.0) ^c^	9,106 (16.4)	< 0.001
**Circumstances** ^d^				
**Interpersonal violence/life stressor**				
Intimate partner violence-related	41 (34.5) ^a,b^	622 (21.9) ^c^	9,637 (17.4)	< 0.001
Family relationship problem ^e^	18 (28.1) ^a,b^	184 (12.0) ^c^	1,480 (5.2)	< 0.001
Crisis during previous or upcoming 2 weeks ^f^	24 (27.3) ^a,b^	354 (16.7) ^c^	3,760 (9.4)	< 0.001
Argument or conflict	31 (26.1) ^b^	947 (33.3) ^c^	22,757 (41.1)	< 0.001
Perpetrator of interpersonal violence in past month ^f^	13 (14.8) ^b^	202 (9.5) ^c^	1,697 (4.2)	< 0.001
Other relationship problem (non-intimate or family) ^e^	4 (6.3)	90 (5.9)	1,473 (5.2)	0.46
Victim of interpersonal violence in past month ^f^	3 (3.4)	54 (2.6)	996 (2.5)	0.84
Jealousy (lovers’ triangle)	3 (2.5)	159 (5.6) ^c^	2,001 (3.6)	< 0.001
Physical fight (2 people, not a brawl) ^e^	1 (1.6) ^b^	90 (5.9) ^c^	4,006 (14.0)	< 0.001
Brawl	1 (0 8)	56 (2.0)	1,223 (2.2)	0.42
**Crime-related**				
Drug involvement	15 (12.6)	513 (18.0) ^c^	7,175 (13.0)	< 0.001
Gang-related	7 (5.9)	235 (8.3) ^c^	3,790 (6.8)	0.01
Drive-by shooting	3 (2.5)	151 (5.3)	2,883 (5.2)	0.41
Hate crime	2 (1.7) ^b^	10 (0.4) ^c^	73 (0.13)	< 0.001
Terrorist attack	2 (1.7) ^b^	4 (0.1) ^c^	6 (0.01)	< 0.001
Walk by assault ^e^	0 (0)	64 (4.2) ^c^	1,632 (5.7)	0.01
**Other**				
Victim(s) killed at work	12 (10.1) ^a,b^	120 (4.2) ^c^	1,580 (2.9)	< 0.001
Random violence ^f, ****^	7 (8.0) ^b^	77 (3.6) ^c^	861 (2.1)	< 0.001
Victim used a weapon	6 (5.0)	257 (9.0) ^c^	3,209 (5.8)	< 0.001
Victim was a bystander	6 (5.0) ^b^	171 (6.0) ^c^	973 (1.8)	< 0.001
Justifiable self defense	5 (4.2)	87 (3.1)	1,584 (2.9)	0.56

^*^ To consolidate information about incidents with > 1 homicide, the authors developed an incident-level dataset with one record per incident (1 or more homicide victims) to describe all victims and suspects in the incident. This avoids overrepresenting data such as incident circumstances and suspect demographics associated with multi-victim homicides by counting these characteristics once per incident instead of once per victim

**All 50 U.S. states, the District of Columbia and Puerto Rico are currently funded to participate in NVDRS, but at the time of this study several of the newer states/jurisdictions had not yet completed a data collection cycle and therefore are not included. States and jurisdictions were first funded to participate in NVDRS in different years. Data for this study comes from the following 37 states/jurisdictions: Maryland, Massachusetts, New Jersey, Oregon, South Carolina, and Virginia (2003–2017); Alaska, Colorado, Georgia, North Carolina, Oklahoma, Rhode Island, and Wisconsin (2004–2017); Kentucky, New Mexico, and Utah (2005–2017); Ohio (2010–2017); Michigan (2014–2017); Arizona, Connecticut, Illinois, Indiana, Iowa, Kansas, Maine, Minnesota, New Hampshire, New York, Pennsylvania, Vermont, and Washington (2015–2017); Hawaii (2015–2016); California, Delaware, West Virginia, the District of Columbia, and Puerto Rico (2017). Illinois, Pennsylvania, and Washington collected data on ≥80% of violent deaths in their state, in accordance with requirements under which these states were funded. Data for California are for violent deaths that occurred in four counties (Los Angeles, Sacramento, Shasta, and Siskiyou)

*** Percentages are based on the number of incidents with known suspect information: mass (126), multiple (2,826), single (55,468). The sum of percentages in columns may exceed 100% because more than one circumstance could have been present per incident

****Random violence is defined as an act in which the suspect is not concerned with who is being harmed, just that someone is being harmed. An example of random violence is an incident in which a person who shoots randomly at passing cars from a highway bridge or opens fire in a crowded shopping mall

^a^Statistically significant difference (*p* < .05) of the prevalence between mass versus multiple homicide

^b^Statistically significant difference (p < .05) of the prevalence between mass versus single-victim homicide

^c^Statistically significant difference (p < .05) of the prevalence between multiple versus single-victim homicide

^d^Unless otherwise specified, percentages are based on incidents with known circumstances: Mass = 119; Multiple = 2,843; Single = 55,416

^e^Circumstances introduced in 2013; denominator adjusted to include only years 2013–2017

^f^Circumstances introduced in 2009; denominator adjusted to include only years 2009–2017

### The victims

Mass homicides had the highest proportion of female victims (52%), and also the highest proportion of child victims; over a quarter of mass homicide victims were younger than 18 years (16% aged < 10 years; 10% aged 11-17 years). Multiple homicides had the next-highest proportion with 14% of victims younger than 18 years (8% < 10 year of age; 6% 11-17 years of age). These proportions were significantly higher than single homicides, which had < 9% victims younger than age 18 years.

Mass homicides had the highest proportion of non-Hispanic white victims (56%), followed by multiple homicides (37%). These proportions were significantly higher than single homicides, which had 29% non-Hispanic white victims. Single homicides had the highest proportion of non-Hispanic black victims (53%), followed by multiple homicides (44%). These proportions were significantly higher than mass homicides, which had 25% non-Hispanic black victims (Table 1).

### The perpetrators

NVDRS had information on 154 suspected perpetrators of mass homicide incidents, 3,651 perpetrators of multiple homicide incidents, and 68,530 perpetrators of single homicides (Table 1). Eighty-nine percent of mass homicide incidents, 82% of multiple homicide incidents, and 74% of single homicide incidents had one or more known suspects (data not shown). Of incidents with a known suspect, 18% of mass, 20% of multiple, and 16% of single homicides had more than one suspected perpetrator (Table 2).

Mass homicide victims were most often killed by strangers (19%), acquaintances/friends (14%), or they were a child (10%) or other family member (9%) of the suspect; 34% of mass homicide victims had an other/unknown relationship to the suspected perpetrator (Table 1). Mass homicide victims were significantly more often killed by strangers and by family members than were other homicide victims.

Across categories, most suspected perpetrators of homicide were males (range: 90–95%), and/or were aged 18-44 years (range: 77–80%). Mass homicides were more likely than other groups to have perpetrators at the older end of this age range, aged 25-44 years (54%), although the differences were nonsignificant in post-hoc analysis.

Mass homicides had a significantly higher proportion of non-Hispanic white perpetrators (44%), than did multiple or single homicides (Table 1).

### The role of mental illness and suicidal behavior

Compared with other groups, a small but significantly higher proportion of mass homicide incidents were thought to be directly related to the suspected perpetrator’s mental illness (mass homicides, 10%; multiple homicides, 5%; single homicides, 2%) (Table 1). Schizophrenia or another unspecified form of psychosis were the leading specific diagnoses noted in these cases across groups (mass homicides, 39%; multiple homicides, 24%; single homicides, 20%).

Suicidal thoughts or behaviors, which included dying by suicide, attempting suicide, or expressing suicidal thoughts, were noted for a significantly higher proportion of perpetrators of mass homicides (30%) compared with the other groups. Multiple homicide perpetrators significantly more frequently also had suicidal thoughts or behaviors compared with single homicide perpetrators (17% vs. 6%).

### Where the homicides occurred

Sixty-seven percent of mass homicides and 59% of multiple homicides occurred exclusively in private homes/apartments (Table 2). These proportions were significantly higher than single homicides. The majority of mass homicide incidents with known suspects that occurred in private residential locations were perpetrated by a current or former intimate partner or family member of the victims (69%; data not shown).

Twenty-one percent of mass homicides and 30% of multiple homicides occurred in a public location (Table 2). For mass homicides, the type of public location was most frequently an ‘other’ specific public location (34%), a street/highway (17%), a bar/nightclub (10%), or a commercial area (14%) (data not shown). Among multiple homicides occurring in public locations, the most frequent types were street/highway (35%), motor vehicle (24%), or other specific location (17%). Two mass homicide incidents (7%), four multiple homicide incidents (< 1%), and 135 single homicides (< 1%) occurred at schools.

Eleven percent of mass homicide incidents, and 10% of multiple homicide incidents had victims killed in more than one location type (Table 2).

### Weapons used and wounds sustained

Across homicide types, fatal injuries were most frequently inflicted using firearms (mass homicides (74%), multiple homicides (82%), single homicides (69%)) (Table 2). Approximately one-third of victims in each group had 3 or more firearm wounds (range: 1-75+; median = 1 for single and multiple homicides, median = 2 for mass homicides; data not shown).

**Fig. 2:**
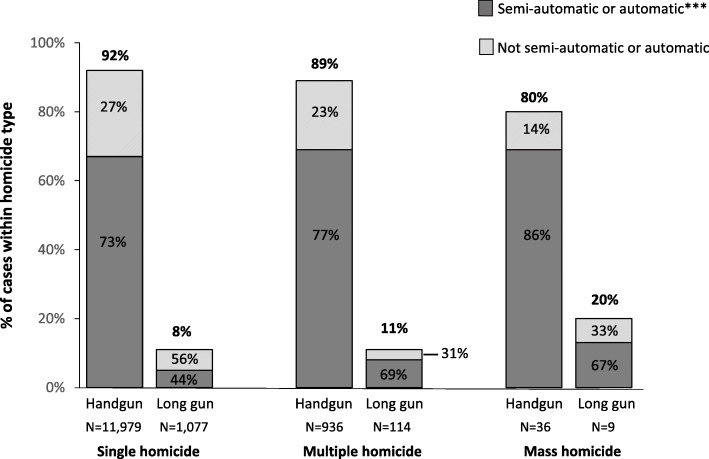
Firearm type* used in single, multiple, and mass homicides, National Violent Death Reporting System, 2003–2017**. *Data presented in this figure reflect firearm homicide incidents where the firearm(s) used had a known “action” type (semi- or fully automatic vs. not semi- or fully automatic) within firearm type (31% single homicides; 51% multiple homicides; 56% mass homicides). The remaining percentage with a known firearm type (handgun vs. long gun) had an “unknown” or “other” action. Across homicide types, an average of 53% of NVDRS incidents have a known firearm type. ** All 50 U.S. states, the District of Columbia and Puerto Rico are currently funded to participate in NVDRS, but at the time of this study several of the newer states/jurisdictions had not yet completed a data collection cycle and therefore are not included. States and jurisdictions were first funded to participate in NVDRS in different years. Data for this study comes from the following 37 states/jurisdictions: Maryland, Massachusetts, New Jersey, Oregon, South Carolina, and Virginia (2003–2017); Alaska, Colorado, Georgia, North Carolina, Oklahoma, Rhode Island, and Wisconsin (2004–2017); Kentucky, New Mexico, and Utah (2005–2017); Ohio (2010–2017); Michigan (2014–2017); Arizona, Connecticut, Illinois, Indiana, Iowa, Kansas, Maine, Minnesota, New Hampshire, New York, Pennsylvania, Vermont, and Washington (2015–2017); Hawaii (2015–2016); California, Delaware, West Virginia, the District of Columbia, and Puerto Rico (2017). Illinois, Pennsylvania, and Washington collected data on ≥80% of violent deaths in their state, in accordance with requirements under which these states were funded. Data for California are for violent deaths that occurred in four counties (Los Angeles, Sacramento, Shasta, and Siskiyou). ***Most firearms within the category “semi-automatic or automatic” were semi-automatic. Across homicide types, 19% of all long guns within this category were classified as fully automatic (range: 17-21%). One mass homicide incident was categorized as being perpetrated with a fully automatic long gun, which was a semi-automatic firearm modified to fire like an automatic firearm

Across categories, most homicides with known firearm types and actions (i.e., firing mechanism) were perpetrated using handguns (range: 80–92%), the majority of which were semiautomatic or automatic firearms (86% of mass, 77% of multiple, 73% of single homicides; Fig. 2). A higher percentage of mass homicides (20%) were perpetrated using long guns (i.e., rifles and shotguns) compared with both multiple (11%) and single (8%) homicides. Most of these were also semi-automatic or automatic firearms (67%).

### Nonfatally shot persons

Over 21% of mass, 16% of multiple, and 8% of single homicide incidents included victims who were nonfatally shot in the incident (Table 2) (range: 1-441). Accounting for nonfatally shot victims, 523 additional incidents in this study can be thought of as “attempted mass homicides” (data not shown). One hundred thirteen were multiple homicides with 1-2+ additional nonfatally shot, 393 were single homicides with 3+ additional nonfatally shot, 9 were suicides with 4+ additional nonfatally shot, and 8 were legal intervention deaths with 4+ additional nonfatally shot.

### Contributing circumstances

#### Intimate partner and family violence

Approximately 35% of incidents of mass homicide were related to intimate partner violence (IPV) (Table 2). These incidents involved violence toward the current or former intimate partner and others present at the scene of the incident. This was significantly higher than the percentage of multiple (22%) and single (17%) homicides that were IPV-related. Multiple homicides were also significantly more frequently IPV-related compared with single homicides. Family relationship problems contributed to mass homicides significantly more frequently as well (28%, vs. 12% of multiple and 5% of single homicides).

#### Other circumstances

Arguments precipitated a substantial proportion of all homicides (mass, 26%; multiple, 33%; single, 41%). A recent or impending crisis was indicated as a precipitating circumstance significantly more frequently for mass homicide incidents (27%) vs. multiple (17%) or single (9%) homicides. Mass homicide incidents also included the highest percentage of workplace homicides, a significantly higher proportion than the other two groups (10%, vs. 4% of multiple, 3% of single homicides).

### How incidents ended

The way the incident resolved (i.e., ended) could be determined in 110 (78%) of mass homicide incidents (data not shown). Among those, the incident most frequently ended with the suspected perpetrator fleeing the scene of the incident (45%), or the death by suicide of the suspected perpetrator (32%). Twenty percent of mass homicide incidents were ended by law enforcement intervention (10% suspect surrendered to law enforcement unharmed; 5% suspect killed by law enforcement; 5% suspect nonfatally injured by law enforcement). In 2 incidents (2%), unarmed citizens disabled the attacker, and no incidents in the data concluded when an armed citizen disabled the attacker. One incident ended with the suspect nonfatally attempting suicide, and one incident ended when the suspect was shot by an unknown person while police were investigating the incident.

## Discussion

While media reports tend to focus on high victim-count incidents perpetrated in public locations, primarily against victims chosen relatively indiscriminately, the results of this analysis show that many incidents of mass homicide actually fit a different profile and have different prevention points. Comparing the characteristics of mass homicides with multiple and single victim homicides also reveals important differences among these groups that may indicate factors that increase the risk that a homicide incident will have a greater number of victims.

The findings reveal important differences across the types of homicide. Mass homicides had a significantly higher percentage of female victims, child victims, and non-Hispanic white victims compared to multiple and single-victim homicides. Mass homicides also had a significantly higher percentage of victims who were a stranger to the suspect but also victims who were connected to the suspect by family relationships. Information on the circumstances surrounding mass homicides shows a significantly higher proportion related to intimate partner violence (IPV) or family relationship problems. The victim characteristics, in conjunction with the information on the circumstances surrounding these incidents, suggest two different prominent types of mass homicide scenarios. In the first scenario, multiple family members including a disproportionate percentage of child homicide victims, are killed during a dispute between intimate partners or other family members. Strategies such as teaching safe and healthy relationship skills(Foshee et al., 2014; Markman et al., 1993; Ruff et al., 2010), engaging men and boys in prevention(Miller et al., 2013), bystander approaches(Moynihan et al., 2015; Coker et al., 2015), and strengthening economic supports for families(Center on Budget and Policy Priorities, 2016; Marr et al., 2015; Sherman et al., 2006; Hartmann et al., 2014; Chatterji & Markowitz, 2005) are effective measures to prevent IPV.(Niolon et al., 2017) The evidence also suggests that policies limiting access to firearms by persons previously convicted of a crime related to IPV or who are under a restraining order for IPV are associated with a reduced risk for intimate partner homicide, overall, and intimate partner homicide by firearm. (Zeoli et al., 2018) Preventing IPV in the first place or stopping IPV from escalating to homicide can save lives, including those of “corollary victims” (Smith et al., 2014) (persons other than the intimate partners themselves who may also be injured or killed in the context of IPV).

The second mass homicide scenario, which includes victims who were strangers to the suspect or connected as an acquaintance/friend, is consistent with incidents of mass violence occurring in schools, workplaces, and other settings. Children and adolescents are the most frequent victims in homicides that take place at schools (Center for Homeland Defense and Security, n.d.), and most school shootings have historically been perpetrated by current or former students.(Everytown for Gun Safety, n.d.-b; National Threat Assessment Center, 2019; Holland et al., 2019) Given the limitations of NVDRS coverage in the years examined, many such incidents were not fully represented in the current study given that school shootings are relatively rare and many occurred in states that were not part of NVDRS at the time. Although the numbers were small, mass homicide incidents also included a significantly higher percentage of victims who were killed at work compared to multiple and single-victim homicides. In mass homicide incidents occurring in schools, workplaces and other settings, prior studies point to early warning signs (e.g., perpetrators threatening violence or harm toward others; perpetrators expressing feeling isolated, bullied, or harassed) and evidence of the perpetrator having other risk factors for violence such as prior involvement in violence as a victim or perpetrator, difficulties at school or home, and suicidal thoughts and behaviors.(Everytown for Gun Safety, n.d.-a; National Threat Assessment Center, 2019) Creating protective environments in schools, workplaces, and other settings by improving organizational policies, practices, and culture can help prevent such incidents.(Everytown for Gun Safety, n.d.-a; Niolon et al., 2017; Bossarte et al., 2006) This includes policies and practices that promote safety and social norms that protect against violence, reduce opportunities for violence, recognize and raise awareness about potential threats or concerning behaviors and facilitate ways to address them, encourage help-seeking behavior, and offer social support and other tangible assistance to persons at risk of harming themselves or others (Niolon et al., 2017; Stone et al., 2017).

A significantly higher percentage of perpetrators of mass homicide compared with perpetrators of multiple or single-victim homicides were noted to be suicidal at the time of the incident. While homicide followed by suicide or a suicide attempt of the perpetrator is a rare occurrence (Barber et al., 2008; Bossarte et al., 2006), it is strongly associated with incidents of intimate partner homicide, especially those committed with a firearm versus another weapon. (Barber et al., 2008; Smucker et al., 2018) Previous research also shows that more than 40% of mass shootings end with the perpetrator dying by suicide.(Everytown for Gun Safety, n.d.-a) In this study, nearly one-third of the incidents ended with the perpetrator dying by suicide. These findings highlight the importance of addressing underlying risk factors for suicide and shared risk and protective factors for interpersonal and self-directed violence. These factors include, for instance, a history of experiencing or perpetrating violence, impulsiveness or poor behavioral control, substance misuse, depression, financial, school, and relationship problems, and a number of community and societal-level social and economic factors. (Wilson et al., 2014) There are many evidence-based strategies that can reduce or protect against these risks. (Niolon et al., 2017; Stone et al., 2017; Fortson et al., 2016)

Firearms, predominately handguns, were the most common weapon used across all types of homicide incidents. A significantly higher proportion of mass homicides were perpetrated with long guns than other homicide incidents, more weapons were used in these mass homicide incidents to inflict fatal injuries than other types of homicide, and a significantly higher percentage of both mass and multiple homicide incidents were perpetrated with semi-automatic firearms compared with single-victim homicides. Previous research shows that access to a firearm in the home is associated with homicide and suicide victimization among household members (Anglemeyer et al., 2014).

A private home or apartment was the most common location in mass and multiple homicides in this study. Previous research also show that most firearms used by youths in school-associated violent death incidents were obtained from their own home or from a friend or relative. (Centers for Disease Control and Prevention, 2003) These studies underscore the importance of identifying effective strategies to help prevent firearms from being used during an interpersonal dispute in the home, an acute suicidal crisis, or from being taken by youth to inflict harm at school or toward others. There is some evidence to suggest that safe storage practices can prevent firearm injury and deaths among children and youth. For example, systematic reviews of the evidence for child access prevention (CAP) laws shows that they are associated with reductions in firearm suicide among youth and unintentional firearm deaths to children less than 15 years of age. Reductions in nonfatal firearm injuries among children under the age of 18 have also been noted, as have reductions in the rate of gun carrying among youth and being threatened or injured with a weapon on school property. (RAND Corporation, 2018; Santaella-Tenorio et al., 2016; Anderson & Sabia, 2018)

With the exception of policies limiting access to a firearm for perpetrators of IPV or under a restraining order for IPV, the evidence for other types of policies on multi-victim homicides (e.g., waiting periods, dealer background checks, bans on the sale of assault weapons and high-capacity magazines, concealed carry laws, extreme risk protection orders) is inconclusive or not known due to a lack of evaluation research or methodological weakness in the studies (RAND Corporation, 2018).

Although mass homicides make up a small percentage of homicides, there are many people living with emotional and physical trauma beyond the numbers reflected by deaths. (North et al., 1997; North et al., 2002; Lowe & Galea, 2017; Fullerton et al., 2019; McKinley et al., 1999; Greenspan & Kellermann, 2002; DiScala & Sege, 2004) While it was not possible to capture the full breadth of impact in our study, when looking even at the data on nonfatal firearm injuries associated with incidents, there were five times as many attempted mass homicides as there were actual mass homicides captured by NVDRS (and this is likely an underestimate based on the validation results for this variable). Exposure to such violence is associated with an increased incidence of a range of short- and long-term physical and negative mental health outcomes, including physical disabilities, depression, and post-traumatic stress disorder (Fullerton et al., 2019; McKinley et al., 1999; Greenspan & Kellermann, 2002; DiScala & Sege, 2004; Lowe et al., 2015), underscoring the importance of preventing these incidents. The benefits of prevention extend to all types of violent assault, as the outcomes referenced above apply across single and multiple victim incidents. The prevention of mass homicides may also have further positive effects due to the potential contagion of these events, particularly in the case of school or other public mass shootings. Previous research has found that there is an increase in other mass shootings following high profile mass shootings for a period of almost 2 weeks. (Towers et al., 2015) Therefore, preventing these incidents can help prevent additional deaths and injuries.

### Limitations

This study has several limitations. First, NVDRS data were not nationally representative at the time of this report, and not all states joined the system at the same time. Many high-profile incidents are thus not included. In future years, however, NVDRS will be able to capture national data as all states, the District of Columbia, and Puerto Rico are now funded to participate. Second, the availability and completeness of data are dependent on successful partnerships among VDRS programs and their partners in vital records, medical examiner/coroner offices, and law enforcement. NVDRS incident data might be limited or incomplete for areas in which these data-sharing relationships are not fully developed. Third, NVDRS collects limited information about suspects and does not provide the level of detail regarding suspect motivations seen in some reports. Abstractors are limited to the information that they receive, and some VDRS programs do not receive detailed law enforcement reports until cases are adjudicated. Fourth, medical and mental health information in NVDRS are not captured directly from medical records but from information contained in the /medical examiner/coroner or law enforcement report based on the information given by witnesses or other informants such as friends or family of the victim and/or suspect. Therefore, some mental health problems may be unknown or misclassified. Further, case narratives vary in completeness in NVDRS; NVDRS coding requirements state that all circumstances endorsed in the system (such as mental illness on the part of the suspect) must be represented in case narratives, but some narratives had very little detail. This variation contributed to the percentage of cases with an unknown/unspecified suspect mental illness type. While it was useful to link data on NVDRS numbers of nonfatally shot persons with other data sources (particularly GVA), GVA only covered 2012 and later. Therefore, the number of incidents in NVDRS identified as having nonfatally shot persons is likely an underestimate. Additionally, NVDRS does not capture instances in which there were no fatalities but where there were a number of nonfatally shot persons.

## Conclusions

This paper examined mass, multiple, and single victim homicides, using a detailed database that combines data from multiple investigative sources, to provide a systematic comparison of the victims, perpetrators, and the circumstances underlying these incidents to inform prevention efforts. The findings complement previous research and show the interconnections between multiple forms of violence. Primary prevention strategies addressing shared risk and protective factors for interpersonal and self-directed violence are key to reducing these incidents and saving lives.

## Data Availability

The dataset analyzed during the current study is available by request via the NVDRS Restricted Access Database application process: https://www.cdc.gov/violenceprevention/datasources/nvdrs/dataaccess.html

## Abbreviations

CDC: Centers for Disease Control and Prevention
FBI: Federal Bureau of Investigation
IPV: Intimate partner violence
NVDRS: National Violent Death Reporting System
